# Pronounced Hypoxia in Models of Murine and Human Leukemia: High Efficacy of Hypoxia-Activated Prodrug PR-104

**DOI:** 10.1371/journal.pone.0023108

**Published:** 2011-08-11

**Authors:** Juliana Benito, Yuexi Shi, Barbara Szymanska, Hernan Carol, Ingrid Boehm, Hongbo Lu, Sergej Konoplev, Wendy Fang, Patrick A. Zweidler-McKay, Dario Campana, Gautam Borthakur, Carlos Bueso-Ramos, Elizabeth Shpall, Deborah A. Thomas, Craig T. Jordan, Hagop Kantarjian, William R. Wilson, Richard Lock, Michael Andreeff, Marina Konopleva

**Affiliations:** 1 Section of Molecular Hematology and Therapy, Department of Leukemia, The University of Texas MD Anderson Cancer Center, Houston, Texas, United States of America; 2 Department of Stem Cell Transplantation and Cellular Therapy, The University of Texas MD Anderson Cancer Center, Houston, Texas, United States of America; 3 Department of Hematopathology, The University of Texas MD Anderson Cancer Center, Houston, Texas, United States of America; 4 Department of Pediatrics, The University of Texas MD Anderson Cancer Center, Houston, Texas, United States of America; 5 C25 Lowy Cancer Research Centre, Children's Cancer Institute Australia for Medical Research, University of New South Wales, Randwick, New South Wales, Australia; 6 St. Jude Children's Research Hospital, Memphis, Tennessee, United States of America; 7 School of Medicine and Dentistry, University of Rochester, Rochester, New York, United States of America; 8 Auckland Cancer Society Research Center, The University of Auckland, Auckland, New Zealand; East Carolina University, United States of America

## Abstract

Recent studies indicate that interactions between leukemia cells and the bone marrow (BM) microenvironment promote leukemia cell survival and confer resistance to anti-leukemic drugs. There is evidence that BM microenvironment contains hypoxic areas that confer survival advantage to hematopoietic cells. In the present study we investigated whether hypoxia in leukemic BM contributes to the protective role of the BM microenvironment. We observed a marked expansion of hypoxic BM areas in immunodeficient mice engrafted with acute lymphoblastic leukemia (ALL) cells. Consistent with this finding, we found that hypoxia promotes chemoresistance in various ALL derived cell lines. These findings suggest to employ hypoxia-activated prodrugs to eliminate leukemia cells within hypoxic niches. Using several xenograft models, we demonstrated that administration of the hypoxia-activated dinitrobenzamide mustard, PR-104 prolonged survival and decreased leukemia burden of immune-deficient mice injected with primary acute lymphoblastic leukemia cells. Together, these findings strongly suggest that targeting hypoxia in leukemic BM is feasible and may significantly improve leukemia therapy.

## Introduction

Self-renewal, differentiation, and mobilization of normal human stem cells (HSCs) are the result of interactions between osteoblastic and vascular microenvironmental domains within the BM [1]–[6]. The osteoblastic niche comprises mainly osteoblasts on the internal surface of the bone and is thought to home quiescent, long-term HSCs. The vascular domain is formed by sinusoidal endothelial cells and is rich in actively proliferating short-term HSCs [7]–[9]. While the importance of the different microenvironmental domains for HSC maintenance is still under investigation, there is evidence for a gradient of decreasing oxygen levels from the vascular to the osteoblastic niche and for the notion that the most primitive HSC are sequestered in a hypoxic microenvironment, implying that low oxygen levels play a fundamental role in the maintenance of normal stem cell function [10], [11]. Quiescent BM cells from regions where blood perfusion is minimal (as determined by low uptake of Hoechst 33342 in vivo) contain a high proportion of long-term repopulating cells capable of serial engraftment [10], [11]. These findings lead us to the hypothesis that a hypoxic microenvironment may support leukemic stem cells (LSC).

It has been shown that hematopoiesis can be increased *ex vivo* by maintaining cell cultures at oxygen levels from 1% to 3%; in this scenario, low pO_2_ levels may prevent oxygen radicals from damaging HSC [12]. Little is known, however, about the role of hypoxia in the maintenance of LSC. Data from a rat leukemia model demonstrated that leukemic cells infiltrating BM reside in markedly hypoxic areas compared to cells in BM of healthy rats [13], [14]. Since hypoxic tumors are in general more resistant to radiation and chemotherapy [15], a hypoxic microenvironment may promote resistance of LSCs, suggesting that the hypoxia itself could be a therapeutic target.

HIF-1α is a key transcriptional regulator of cellular response to hypoxia. HIF-1α subunits are normally degraded by the protein encoded by the von Hippel-Lindau tumor suppressor gene *VHL* (pVHL) in the presence of oxygen but are stabilized under conditions of hypoxia. Activation of the HIF-1α signaling pathway induces a vast array of gene products controlling energy metabolism, glycolysis, angiogenesis, apoptosis, and the cell cycle [16], [17]. Recent genetic studies have shown that normal HSCs stabilize HIF-1α protein critical for maintaining cell cycle quiescence [18] and utilize glycolysis instead of mitochondrial oxidative phosphorylation [19]. HIF-1α is overexpressed in a broad range of human cancer types and increased levels of HIF-1α activity are often associated with increased tumor aggressiveness and therapeutic resistance [16]. HIF-1α may be clinically significant in acute leukemia as the protein has been shown to be overexpressed in clusters of leukemic cells in the BM of pediatric ALL patients [20].

Novel strategies to target HIF-1α in tumors are being developed to counter hypoxia's pro-survival effect on cancer cells [21]–[23]. These strategies include inhibition of signal transduction pathways that have an impact on HIF-1α and direct inhibition of HIF-1α expression or activity by antisense or small molecules [21], [24]. A disadvantage in focusing on HIF-1α as a chemotherapeutic target is that there are other HIF subunits (e.g HIF-2α) that are functional during hypoxia and the importance of the various subunits depends on which cell type is involved [25], [26]. A second problem with targeting HIFs is that they are regulated in a complex manner by many factors other than oxygen concentration, and can also be stabilized by mutation of *VHL*, so not all hypoxic cells express HIFs and conversely not all HIF-expressing cells are hypoxic. While HIFs may be valid targets in their own right, bioreductive prodrugs potentially provide a more direct mechanism for sensing and eliminating hypoxic cells. A more effective means of therapy would involve the use of an agent that is inert in normoxic conditions but is converted to a lethal form in the absence of oxygen. Such agents do exist and include tirapazamine and AQ4N [23], [27]. Among the agents currently under investigation, PR-104 is one of the more promising candidates. PR-104 is a phosphate ester that is rapidly hydrolyzed *in vivo* to the corresponding alcohol PR-104A. Under extremely low oxygen concentrations, PR-104A is reduced mainly to the amine and hydroxyl-amine nitrogen mustards that induce DNA cross-linking in hypoxic cells [28], [29].

In this study, we evaluated the role of hypoxia in the leukemic microenvironment, with particular attention to its relation with chemoresistance of leukemic cells. We show for the first time that HIF-1α is expressed in BM biopsies of ALL patients at diagnosis and that hypoxia areas are vastly expanded in leukemic BM, in which it contributes to microenvironment-mediated chemoresistance. Hypoxia-activated prodrug PR-104 demonstrated remarkable single agent activity in *in vivo* ALL models, indicating feasibility of exploiting hypoxia in the setting of acute leukemias. Altogether, these findings suggest that hypoxia plays a role in microenvironment-mediated chemoresistance in ALL and strongly suggests that hypoxia-targeting agents may be effective new tools in the treatment of ALL.

## Results

### Expansion of hypoxic niches within leukemic BM

**Figure 1:**
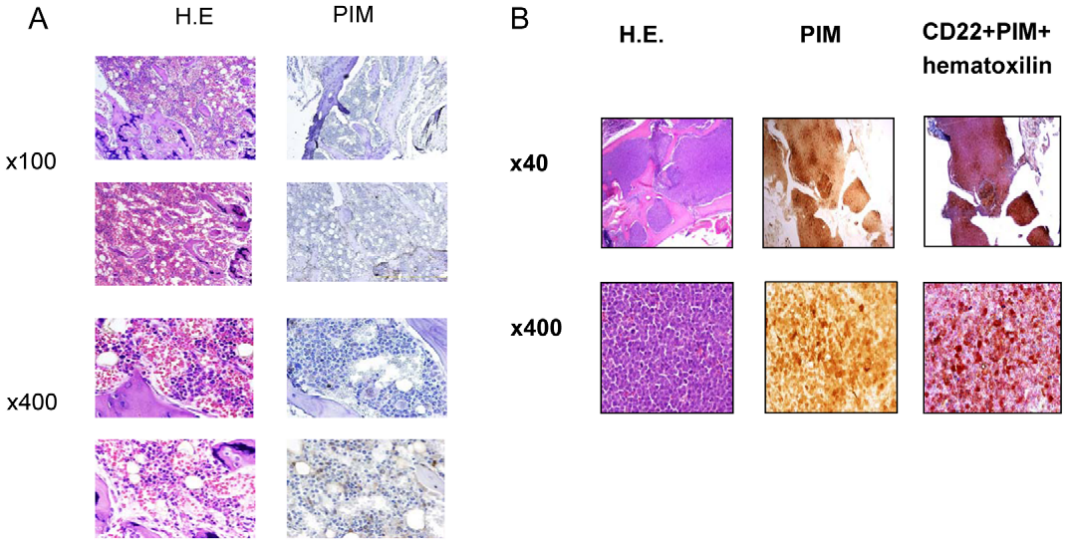
Expansion of the hypoxic BM niche in an ALL xenograft model. Representative images from one healthy control mouse (two different BM fields, A) or mice transplanted with CD22-expressing leukemic cells from an infant with MLL-rearranged B-lineage ALL (B). After 52 days, mice were injected with pimonidazole (PIM) 3 hrs prior to sacrifice. Areas of hypoxia were detected by PIM antibody, and leukemic cells by anti-CD22. Original magnification is shown next to each panel.

**Figure 2:**
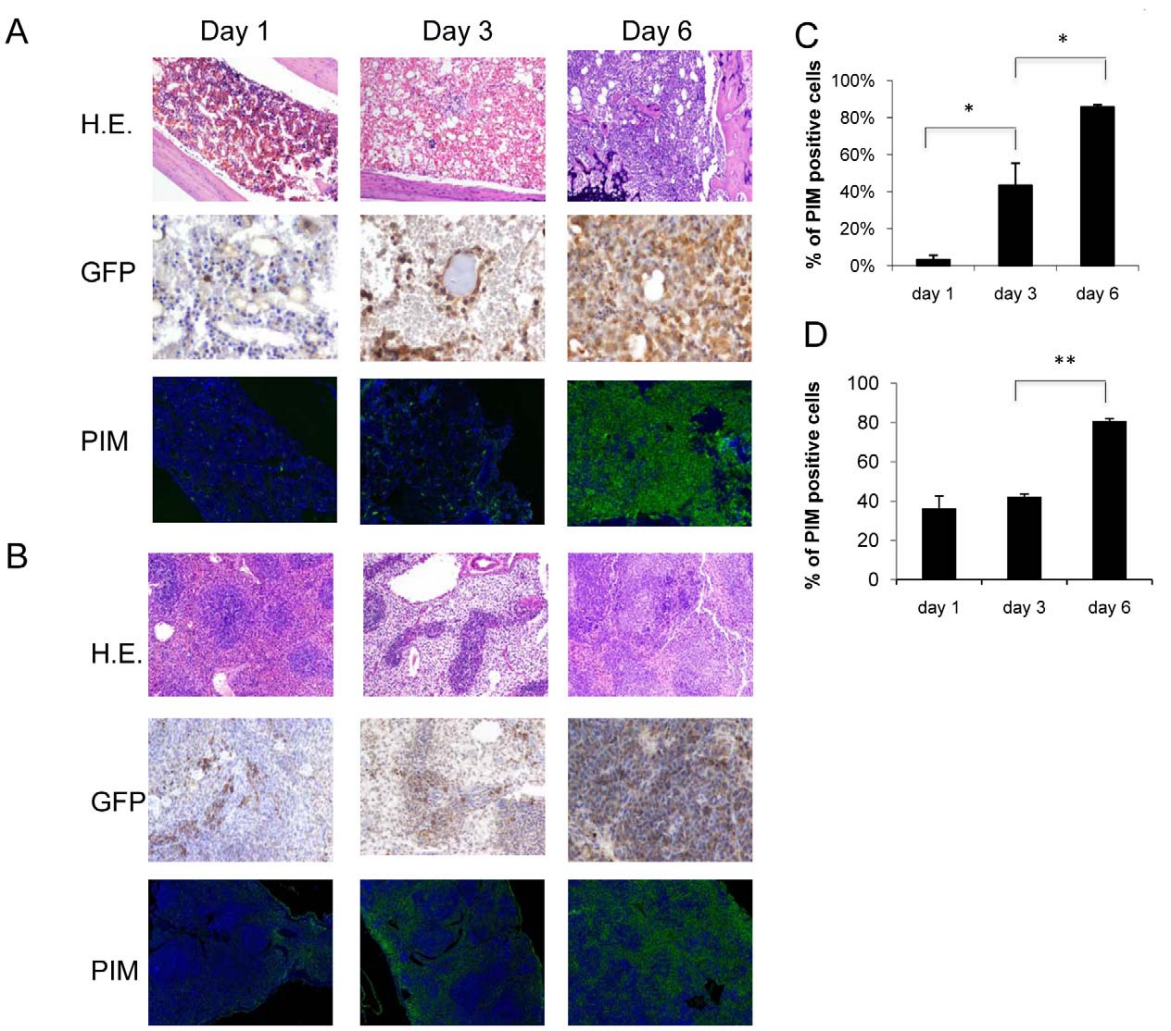
Expansion of the hypoxic BM niche in a syngenic model of blastic phase CML. 1×10^7^ GFP/YFP labeled cells expressing the oncogenes *BCR/ABL* and *Nup98* were FACS-sorted and transplanted into irradiated (4.5GY) C57B16/J mice. At the indicated time-points, mice were injected with pimonidazole 3 hrs prior to sacrifice. Areas of hypoxia were detected by PIM, and leukemic cells by anti-GFP in BM (A) or spleen (B). (C) and (D) Quantification of PIM positive cells in BM and spleen, respectively by CRi spectral imaging and Inform software analysis (at least 3 mice per group were analyzed). Original magnification, ×100. *P<0.05; **P<0.01.

To examine the extent of hypoxia in leukemic vs. normal BM, we determined the levels of hypoxia in the BM microenvironment of control NOD/Scid/IL2Rγ-KO (NSG) mice and mice xenografted with human or murine leukemic cells. To evaluate hypoxia, we utilized the specific chemical marker of hypoxia pimonidazole (PIM), a 2-nitroimidazole compound that under hypoxic conditions is metabolized and forms stable adducts inside the cells that can then be detected with a specific antibody. First, we validated specificity of PIM staining in ALL cells exposed to different oxygen levels. In leukemic cell lines REH and NALM6, PIM staining measured by flow cytometry was positive at 1% oxygen and further increased at 0.1% oxygen, indicating specific binding of this nitro-imidazole compound to hypoxic ALL cells (Fig. S1). We next utilized PIM immunohistochemistry (IHC) to assess levels of hypoxia in the BM of immunodeficient mice used as recipient for human leukemia engraftment. As shown in Figure 1A, PIM staining showed few discrete areas of positivity in control mice not injected with leukemic cells. In contrast, there was a great expansion of the hypoxic areas in the BM of the NSG mice injected with primary human B-ALL cells, with a uniform pattern of PIM positivity that co-localized with CD22^+^ staining of ALL cells (Figure 1B). To examine the role of hypoxia using immune competent mice, we made use of a syngenic model of blastic phase chronic myelogenous leukemia (CML) whereby C57Bl6/J mice were injected with murine bone marrow cells expressing the BCR-ABL and Nup98 translocation products, and green fluorescent protein GFP [30]. In this model, the progression of leukemia (by GFP staining) and development of hypoxia (using PIM as above) in the BM and spleen was followed over time (Figure 2). Twenty hours after injection of the cells, there were very few cells showing positive signal in both tissues analyzed. Three days after cell injection GFP positivity became apparent, with several areas of PIM positivity. By day 6 after injection, both BM and spleen exhibited vast areas of PIM positivity (Figure 2). This rapid progression of leukemia was accompanied by notable changes in the BM vasculature revealed by lectin labeling of blood vessels (Fig. S2). Modest decrease in the microvessel density was observed in BM from leukemia-bearing mice compared to healthy controls. In addition, the vasculature in the leukemic BM appeared morphologically different than that in the normal BM, with elongated and distorted vessels. These findings indicate that expansion of hypoxia in the in vivo murine models of leukemia is accompanied by a loss of vessel integrity with increased leukemic cell burden.

**Figure 3:**
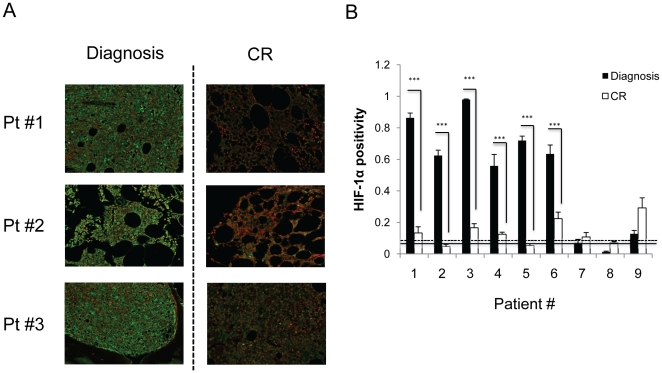
Expansion of the hypoxic BM niche in primary leukemia samples reverted following complete remission (CR). HIF-1α was detected by IHC in BM biopsies from 9 ALL patients at diagnosis and at CR after induction chemotherapy. (A) Representative images from 3 patients are shown. Original magnification, ×500. (B) Quantification of HIF-1α expression using Cri Inform software. A threshold was established based on the scores of three normal BM samples (continuous line represents average HIF-1α and dashed line correspond to SD for these 3 samples, respectively) CR; complete remission *** P<0.0001, error bars SEM.

To validate these findings in human BM specimens, we evaluated expression of HIF-1α, as an established marker of hypoxia, by quantitative immunohistochemistry using CRi Nuance spectral imaging system. BM biopsy specimens from 9 ALL patients at the time of diagnosis and after induction chemotherapy upon recovery of normal hematopoiesis, were stained with HIF-1α antibody (Figure 3). As controls, HIF-1α immunostaining was assessed in BM sections from 3 normal donors. HIF-1α was expressed at very low levels in the normal bone marrow (Figure S3A). In agreement with our observations in mice, HIF-1α was strongly positive in 6 of the 9 BM biopsies obtained from ALL patients at the time of diagnosis (see examples in Figure 3). In contrast, HIF-1α signal was expressed in only a few cells in the paired BM samples obtained after patients have achieved complete remission (CR). Importantly, HIF-1α was expressed not only in leukemic blasts, but also in the surrounding stromal cells (Figure S3B), indicating expansion of the hypoxic niches. In 3 out of the 6 patients the levels of HIF-1α at CR were very close to those observed in normal BMs (indicated by the continuous line on Fig. 3B). These results suggest stabilization of HIF-1α protein in BM infiltrated with leukemic cells and possible reinstitution of higher oxygen levels upon restoration of normal hematopoiesis.

**Figure 4:**
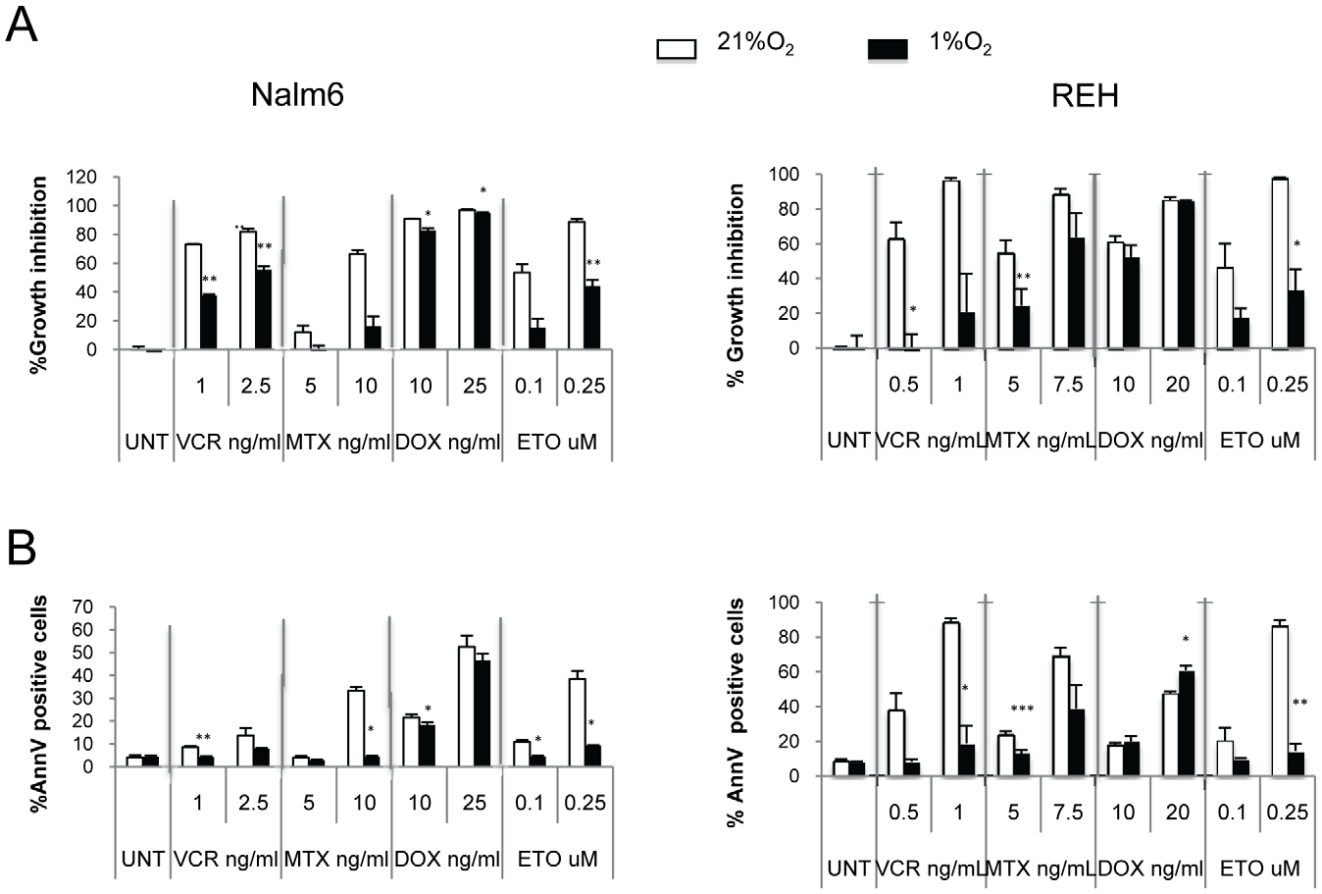
Hypoxia promotes resistance of leukemia cell lines to chemotherapy. Cytotoxic effect of chemotherapy under normoxic and hypoxic conditions. Nalm6, REH ALL cells were treated with various chemotherapy drugs for 48 hours at 21% or 1% O_2_. Percentage of growth inhibition was calculated based on cell number. A and B: cell number and percentage of Annexin V–positive cells were determined by FACS. Error bars, SEM. * P<0.05; ** P<0.01; *** P<0.001.

To determine whether hypoxia influenced the sensitivity of leukemic cells to chemotherapy, *in vitro* experiments were performed to compare the effects of standard chemotherapeutic agents at different oxygen levels. REH and Nalm6 B-lineage ALL cells were exposed to various chemotherapy agents (vincristine, methotrexate, doxorubicin, or etoposide) for 48 hours under either normoxic (21% O_2_) or hypoxic (1% O_2_) conditions. Effects on viability and cell death were determined by flow cytometry. At 21% O_2_, all drugs induced cell killing in a dose-dependent manner. By contrast, a significant decrease in growth inhibition (the hypoxic drug values were normalized to the hypoxic control) and apoptosis induction was noted in REH and NALM6 cells treated with vincristine, methotrexate, and etoposide under hypoxic conditions (Figure 4A). A minimal effect was observed for doxorubicin, however not as impressive as the hypoxia induced-chemoprotection against the other drugs tested.

### In vitro hypoxia-selective cytotoxicity of PR-104

**Figure 5:**
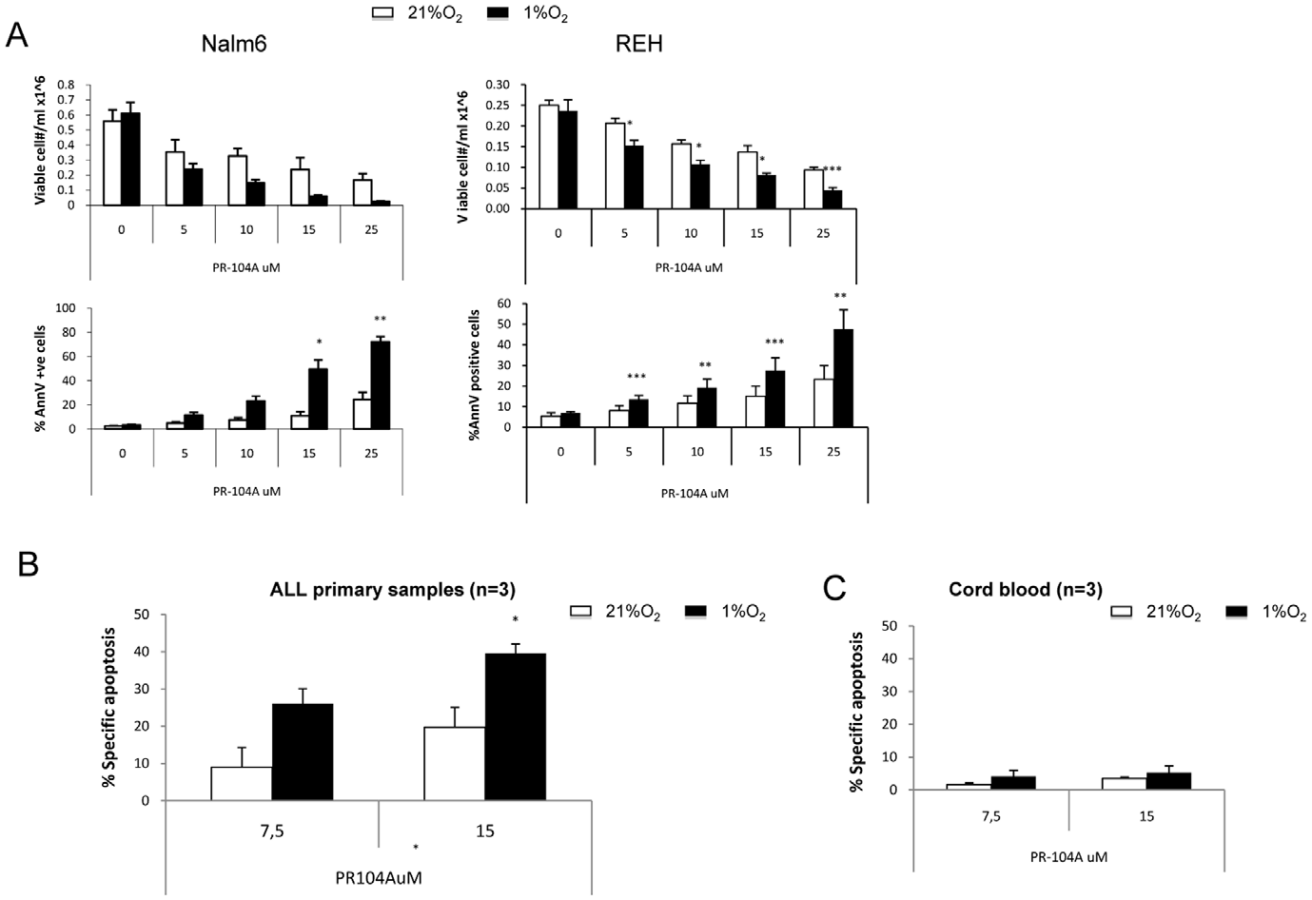
In vitro hypoxia cytotoxicity of PR-104A. (A) B-cell ALL cell lines Nalm6 and REH B-ALL were treated with PR-104A under normoxic or hypoxic conditions for 6 hours. Cell death, measured as percentage of Annexin V (AnnV)–positive cells and cell number were measured 48 hours later by FACS. (B) and (C) Three primary ALL samples (B) or three control CB (C) were treated with PR-104A at normoxic or hypoxic conditions for 6 hours. Cell death, measured as percentage of Specific Apoptosis [% Specific Apoptosis = (%AnnV sample-%AnnV control)/(100-%AnnV control)*100] were measured 24 hours later by FACS. *P<0.05; **P<0.01; ***P<0.0001.

Given the evidence that hypoxia mediates chemoresistance of leukemic cells, we sought to exploit the hypoxic microenvironment of leukemia by utilizing hypoxia-activated pro-drugs. We first determined the hypoxia selectivity of DNA-cross linking agent PR-104 against leukemia cell lines. To examine PR-104 cytotoxic activity, cells were treated with the alcohol form of the drug (PR-104A) for 6 hours at 21% or 1% O_2_, and cell death was assessed by FACS determination of Annexin V staining at 48 hours. Concentrations were selected based on the therapeutic levels achievable in humans, which provides a plasma AUC of ∼40 µM-h at the MTD of 1100 mg/m2 in patients with solid tumors [31]. In REH and Nalm6 pre-B ALL cells, PR-104A induced greater growth inhibition at 1% O_2_ compared to 21% O_2_ culture conditions (Figure 5A).

While PR-104A toxic effect was maximal under hypoxia, there was some degree of drug-induced cell death in both ALL cell lines even in normoxic conditions. Even though activation of PR-104 requires very low oxygen levels, Guise et al. reported that PR-104 can also be activated by the aldo keto reductase enzyme prostaglandin F synthase, 17b-hydroxysteroid dehydrogenase type V (AKR1C3) independent of oxygen level [32]. Since we observed cytotoxicity, although diminished, at ambient oxygen levels, we evaluated the presence of the AKR1C3 enzyme in the three cell lines tested. As shown in the Western blot in Figure S4, when compared to the SKOV3 cells used as positive control, Nalm6 did not express detectable levels of the enzyme, while REH cells did. This observation is consistent with the fact that REH exhibited higher sensitivity to PR-104A in normoxia than Nalm6 cells.

PR-104A activity was next tested in primary ALL samples. Figure 5B shows that PR-104A also had higher activity against primary leukemia cells at 1% compared to 21%O_2_. However, in primary samples PR-104A induced apoptosis, albeit at less extent, at 21% O_2_, which led us to check the levels of AKR1C3 in samples from patients as well as normal donors. We found that patient samples have in general higher levels of AKR1C3 than CD34^+^ cells isolated from normal donors, in agreement with high AKR1C3 message levels reported by Birtwistle et. al. for AML patients [33] (Fig S4B and C). AKR1C3 levels were variable, but one third of the ALL samples showed high levels suggesting that high AKR1C3, in addition to hypoxia, might also be a target in some leukemias. This observation could explain the PR-104A sensitivity observed at 21% O_2_ and also provides an additional rationale for the use of PR-104, since this drug would be activated not only under hypoxic conditions but also selectively by leukemia cells expressing high levels of the enzyme. We also tested the activity of PR-104A against mononuclear cells isolated from cord blood samples, used here as a normal control (Figure 5C). PR-104A did not induce apoptosis in these cells either at 21% or at 1 %O_2_ (N = 3).

### In vivo PR-104 antitumor activity

**Figure 6:**
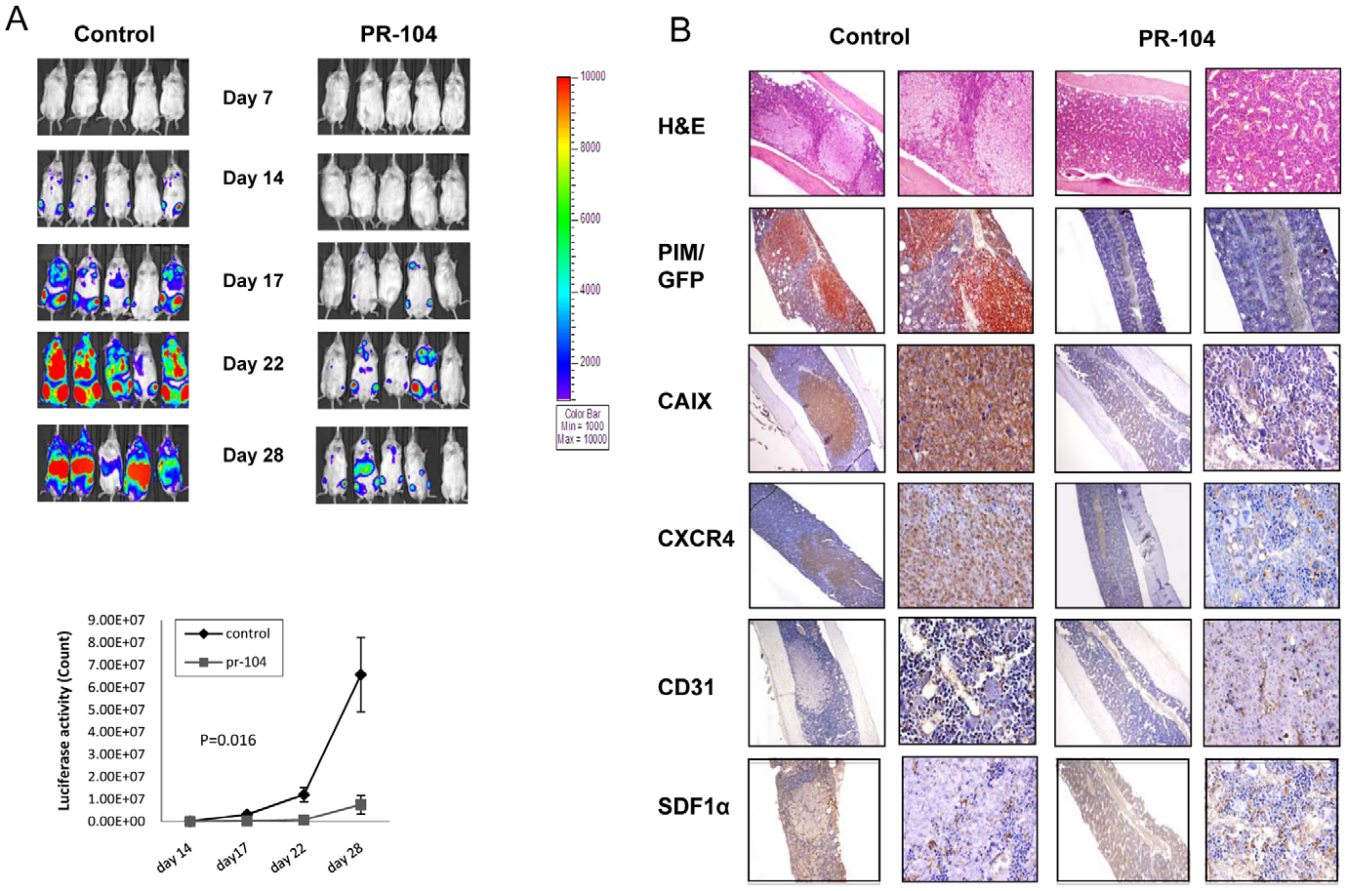
PR-104 administration resulted in decreased tumor burden and hypoxic areas in a Nalm6/GFP ALL model. NSG mice injected with Nalm6-GFP/Luciferase cells were treated with PR-104 starting on day 3 after injection (250 mg/kg, i.p. 3 times a week for 2 weeks). PIM was administered 3 hours prior to sacrifice. (A) Luciferase activity in PR-104-treated and control mice over the course of the experiment. Bottom graph: Luciferase quantification of control and PR-104 treated mice. (B) BM sections from control and treated mice stained for the hypoxia markers PIM and CAIX and for GFP, CXCR4, CD31 and SDF1α. Original magnification, ×100 and ×400 (H&E) ×40 and ×400 (PIM/GFP, CAIX, CXCR4, CD31 and SDF1α).

To test the *in vivo* efficacy of PR-104, several leukemia models were employed. Our first xenograft model focused on using Nalm6 cells transduced with Luciferase. NSG mice injected with Nalm6-GFP/Luciferase cells were treated with PR-104 starting on day 3 after injection (250 mg/kg, i.p. 3 times a week for 2 weeks), and leukemia progression was monitored by luciferase imaging. While control animals showed luciferase positivity indicating detectable tumor burden on day 14 after injection, none of the PR-104-treated mice had a positive signal (Figure 6A). Twenty two days after injection, all control mice were strongly positive for luciferase activity while the treated animals showed much smaller positive areas, indicating that PR-104 inhibited the growth of Nalm6 cells *in vivo*. Remarkably, on day 28 (when mice were sacrificed) one mouse from the PR-104 treated group remained leukemia-free while all the mice in the control group exhibited high levels of luciferase activity. Before the mice were sacrificed, PIM was injected to evaluate BM hypoxia. As shown in Figure 6C, the BM of control animals were predominantly infiltrated by GFP^+^ leukemia cells, which colocalized with PIM adducts, whereas the PR-104-treated mice had very few GFP^+^ cells and only small areas of PIM staining. Expression of the HIF-1α targets CAIX and CXCR4 correlated very closely with that of PIM, confirming the expansion of hypoxic zones in the untreated leukemic BM and inhibition of this process upon PR-104 treatment. No significant differences were seen in the expression levels of stroma-derived factor 1α (SDF-1α). Although the microvessel density assessed by CD31 staining did not differ significantly between control and treated mice, vessels appeared tortured and compressed in leukemic as compared to normal BM.

**Figure 7:**
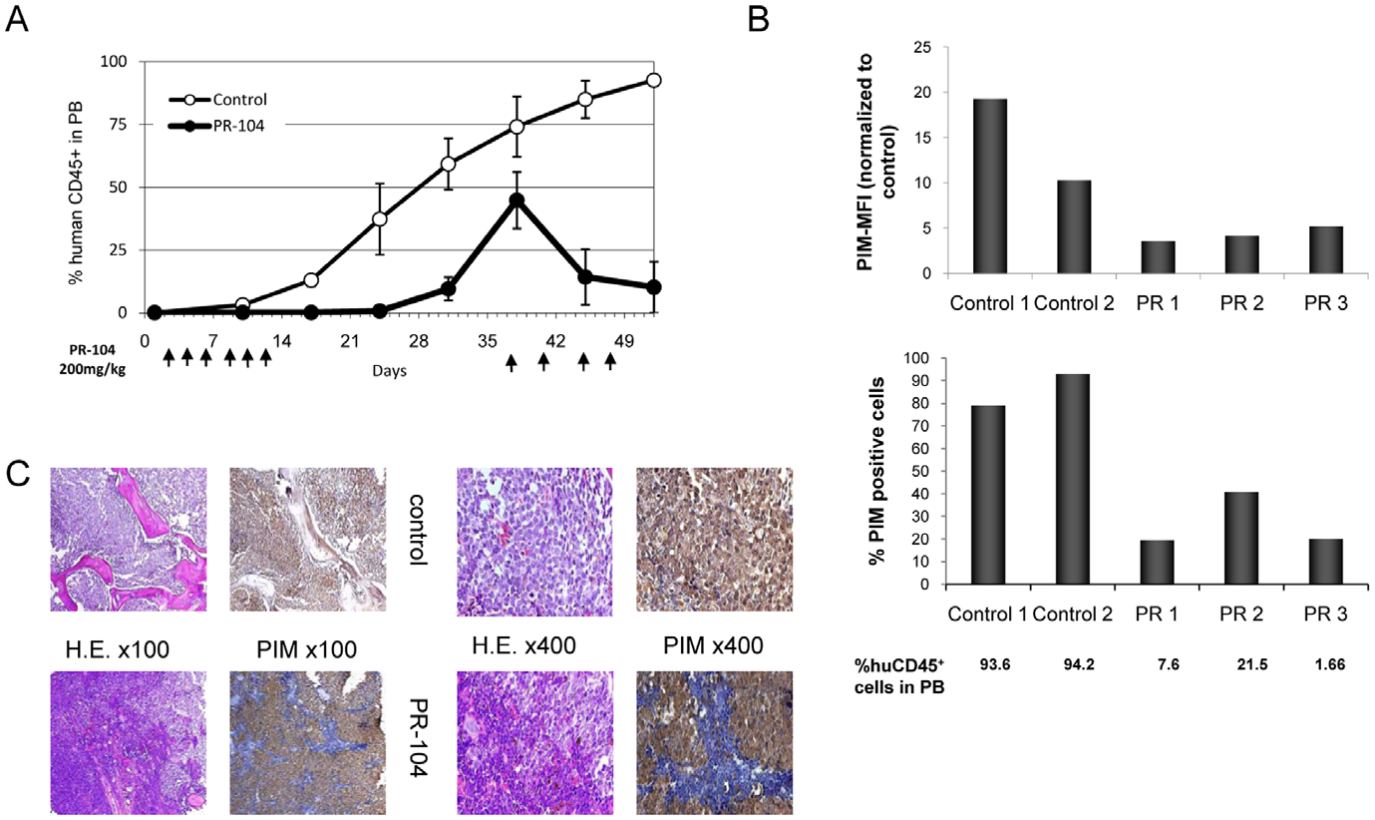
PR-104 induced decrease of circulating CD45^+^ cells and of hypoxia in NSG mice injected with a primary B-lineage ALL sample. (A) Time course of changes in percentage of circulating CD45^+^ cells determined in peripheral blood by FACS (n = 9/group). Arrows at the bottom indicate days when PR-104 was administered. (B) PIM in BM flushes from two control and three PR-104-treated mice determined by FACS. PIM was administered 3 hour prior to sacrifice on Day 52. PIM MFI is plotted as a ratio between the sample MFI/unstained control MFI. Bottom panel: percentage of PIM positive cells. The % of human CD45 positive cells in peripheral blood (PB) is indicated for each mouse. (C) Pimonidazole adducts and H&E staining in BM sections from control and PR-104-treated mice determined by IHC. Original magnification is indicated.

To examine the efficacy of PR-104 in killing primary ALL cells *in vivo*, NSG mice were injected with leukemic cells from an infant with MLL-rearranged B-lineage ALL. Leukemia burden was determined by the percentage of human CD45^+^ cells circulating in peripheral blood. In control mice, the percentage of circulating CD45^+^ cells increased steadily, reaching 93±2.3% on day 52 (Figure 7A). In contrast, CD45^+^ cells remained undetectable in treated mice while they received the drug and up to a week later. Percentage of circulating CD45^+^ cells then increased but decreased significantly on re-initiation of treatment (from 45±15% on day 38 to 10±10.2% on day 52). PIM was administered to the control and treated mice before they were sacrificed, and cells containing PIM adducts and therefore hypoxic were determined in BM flushes by flow cytometry. In control mice (n = 2), PIM intensity ratios in the BM flushes (normalized to the intensity for the same cells but without PIM antibody) were 20 and 10, respectively, and the percentage of PIM positive cells was 79 and 93%, respectively. In contrast, in the treated mice (n = 3), PIM intensity ratios decreased to 4 and 5, and the percentage of PIM positive cells was 20% and 40%, respectively, indicating that the level of hypoxia in the BM of these mice was significantly lower than in controls (Figure 7B). The extent of hypoxia in control vs. treated mice was additionally confirmed in BM sections from both groups by histochemical detection of PIM adducts after treatment (Figure 7C). In control mice, the BM was fully PIM positive, which correlated with the higher ratio of PIM intensity detected in BM flushes by flow cytometry. In treated mice, on the other hand, the BM had hypoxia-free zones (blue staining in Figure 7C) in accordance with the lower ratios of PIM intensity detected in BM flushes from these mice and also with the decreased percentage of circulating CD45^+^ cells.

**Figure 8:**
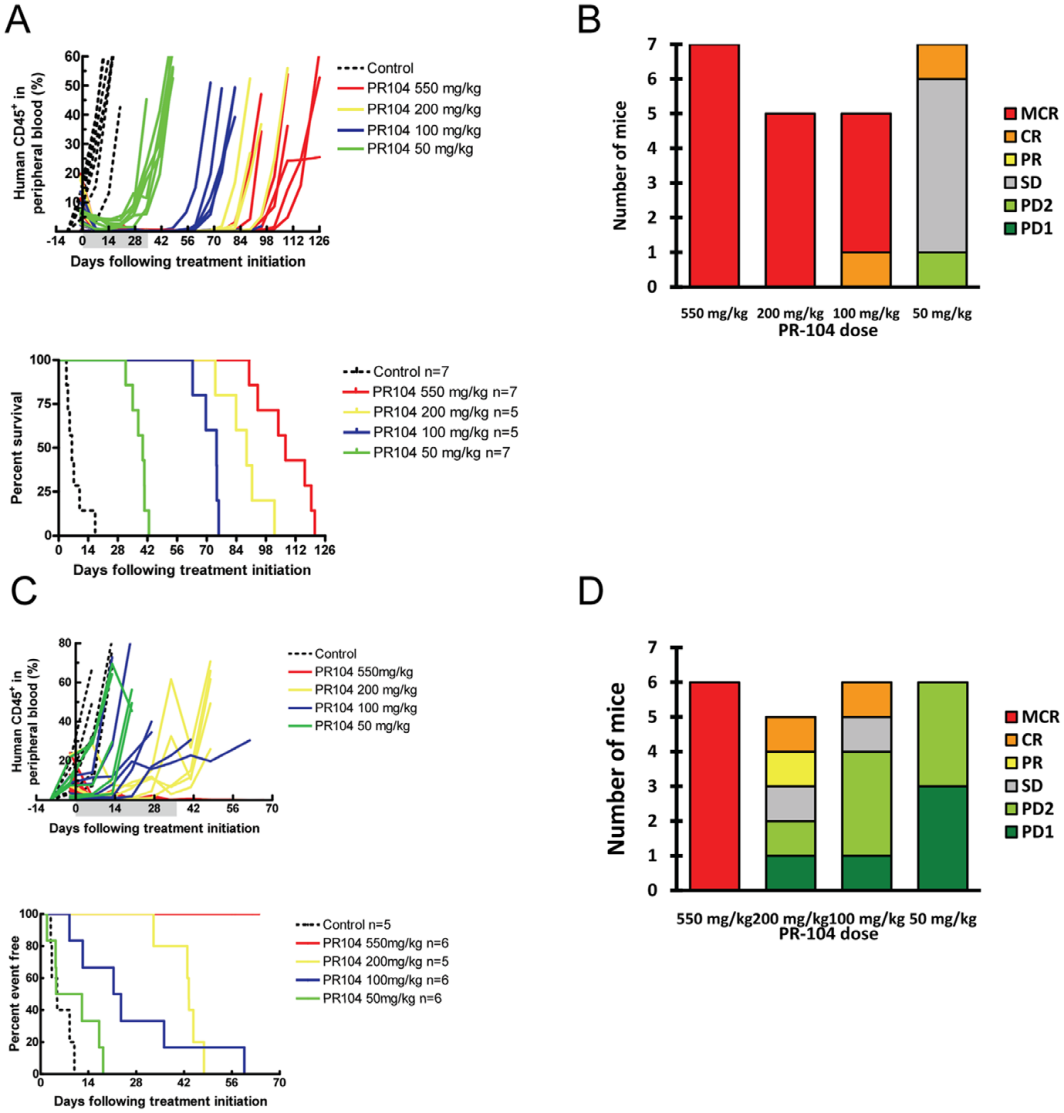
Dose-response efficacy of PR-104 against ALL-8 and ALL-19 in vivo. (**A and C**) The responses of NOD/SCID mice engrafted with ALL-8 (A) or ALL-19 (C) xenograft to treatment with PR-104 at decreasing doses are represented as percentages of human CD45^+^ cells in the peripheral blood of individual mice over time. PR-104 was administered once a week for 6 weeks via i.p. injection; the shaded area corresponds to the duration of treatment. Kaplan-Meier plots of EFS, from which LGD was calculated (difference between median EFS of treated and control mice) and compared using the logrank test. The corresponding values are shown in **Table S1**. (B and D) Distribution of objective response measures for ALL-8 (B) or ALL-19 (D) engrafted individual mice at each dose of PR-104 tested. MCR, maintained complete response; SD, stable disease; PD, progressive disease.

Next, anti-leukemia effects of PR-104 were evaluated in NOD/Scid mice injected with cells from two pediatric ALL patients propagated *in vivo* (referred to as “xenografts”, ALL-8 and ALL-19 [34]). In these studies, PR-104 was administered to several groups of mice in a dose-dependent manner over clinically relevant doses (200, 100 and 50 mg/kg). The maximum tolerated dose (MTD) of 550 mg/kg dose was also included for comparison. Compared to vehicle control, PR-104 significantly delayed progression of ALL-8 at all doses tested, resulting in significantly increased event-free survival (EFS) of mice in all treatment groups (Figure 8A, Table S1). The efficacy of PR-104 was dose dependent. The estimated leukemia growth delay (LGD) values, calculated according to established methodology [34], [35], ranged from 100.9 to 33.5 days for the highest and the lowest dose of PR-104, respectively. In ALL-8 xenografted mice, the three highest doses of PR-104 produced objective responses, all of which were maintained complete responses (MCR), whereas treatment with the lowest dose resulted in stable disease (Figure 8B). Compared to vehicle control, PR-104 significantly delayed leukemia progression of ALL-19 at three of four doses tested, resulting in significantly increased EFS of mice in these treatment groups (Figure 8C, Table S1). Estimated LGD values ranged from >54.6 to 3.4 days for the highest and the lowest dose of PR-104, respectively. Only treatment at the highest dose of the drug resulted in the objective response of MCR (Figure 8D).

Finally, the anti-leukemia effects of PR-104 were tested in against a primary xenograft using human AML cells. PR-104 at 250 mg/kg administered on day 72 after injection of NSG mice with primary refractory AML cells resulted in a statistically significant prolongation of survival when compared to control mice that did not receive therapy (median survival time (MST) of control and PR-104 treated mice 86 and 169 days, respectively, n = 5 mice/group; *P* = 0.026, not shown). During the course of the treatment, leukemia was assessed as circulating human CD45 positive cells (Figure S5A): while there was persistence or gradual increase in the percentage of circulating human CD45 positive cells in the PBS-treated mice, this population remained undetectable in PR-104-treated mice. After treatment, leukemia engraftment was evaluated in different tissues by staining for CD45^+^. Whereas in control mice CD45^+^ cells had infiltrated lung, liver, bone, and spleen, in treated mice these cells were almost undetectable in the tissues examined (Figure S5B) indicating that PR-104 treatment not only prolonged survival but also resulted in marked reduction of tissue infiltrating leukemia cells. Assessment of hypoxia levels in the BM by PIM staining (Fig S5C) showed that the BMs of control mice were extensively hypoxic, which contrasted with the smaller, more discrete hypoxic areas observed in the BM of treated mice.

## Discussion

Using the hypoxia marker pimonidazole, we have shown in several *in vivo* models of human and murine leukemia that the BM becomes highly hypoxic in advanced stages of the leukemia process. Furthermore, BM from ALL patients stained for HIF-1α showed very strong positivity at diagnosis that was impressively reduced or eliminated when the patients achieved CR. This is the first report indicating that the expansion of hypoxic areas is one of the essential characteristics of the leukemic microenvironment. The mechanism responsible for this expansion remains elusive at this point, but one possible explanation is that accumulation of leukemic blasts in the BM leads to increased oxygen consumption, thus lowering steady-state oxygen concentrations. Time-course studies using a blast crisis CML model support this hypothesis, clearly demonstrating that evolving hypoxic areas are associated with leukemia progression. Although not thoroughly investigated in our studies, it is also likely that leukemia propagation is associated with derangements and non-functionality of vascular architecture which might contribute to progressive hypoxia despite stimulation of angiogenesis through HIF-1α. This notion is supported by findings by Schaefer et al. [36] demonstrating time-dependent alterations in the microvasculature in the in vivo leukemia models, with the early angiogenic wave followed by decreased vessel density and reduced tissue perfusion at the late stages of leukemia. We have observed that not only leukemic cells but also the surrounding stroma expresses HIF-1α (Fig. S3B), implying that hypoxia is an intrinsic property of the leukemia microenvironment. While HIF-1α expression is frequently promoted in normoxic tumor cells, for example through oncogenic stimuli, generating molecular signatures resembling hypoxic response in the absence of hypoxia (coined as “pseudohypoxia” [37]–[39]), hypoxic marker Pimonidazole can only be metabolized and form intracellular adducts at low oxygen concentrations, thus reflecting true hypoxia as opposed to pseudohypoxia. In a recent report, Hu et al. investigated the BM microenvironment in multiple myeloma models and observed vast hypoxic expansion (revealed by PIM staining), while naïve mice did not exhibit PIM positivity [40]. In agreement with this, Colla et al showed that BM hypoxia and high HIF-1α expression is a characteristic of multiple myeloma patients [41]. Therefore, a hypoxic microenvironment may be a common phenomenon in bone marrows with expanding tumor cell populations. We also noted only infrequent HIF-1α positive cells in normal BMs supporting the notion that under physiological conditions the BM harbors only discrete areas of hypoxia.

Our exploration of the role of hypoxia in leukemic cells showed that, *in vitro*, 1% O_2_ levels conferred resistance against selective chemotherapeutic agents in the ALL cell lines tested. The mechanism responsible for this effect was not addressed in the present work. However, a potential candidate is HIF-1α given its role as master regulator of the hypoxic response. Many known HIF-1α targets could mediate the protection conferred by hypoxia, and a number of those have been validated as chemoresistance factors in leukemias (i.e., MDR-1, Nur-77, CXCR4 and others) [16], [42]. In this regard, we have previously reported that hypoxia increases CXCR4 expression leading to increased migration and survival of leukemic cells [43]. HIF-2α is another HIF family member induced by chronic hypoxia and expressed at high levels in primary tumors and their metastases [25], [44]. HIF-2α targets overlap with but seem to be different from those regulated by HIF-1α [26], [45], [46]. Which HIF-1α targets mediate hypoxia-induced chemoprotection in leukemia cells and whether there is any contribution from HIF-2α requires further investigation. In preliminary studies, HIF-2α was not found to be expressed in the ALL cell lines used for this study. Furthermore, HIF1α-independent mechanisms have been described as mediating hypoxia responses in various systems. Among them, NF-κB is of particular interest given that it can be induced by hypoxia and can regulate important pathways including cell proliferation, angiogenesis, metastasis and survival [47]. Its importance in hematological malignancies is becoming clearer [48], [49] and in fact, two groups [50], [51] recently demonstrated that modulation of the NF-κB pathway sensitized leukemic cells to chemotherapy and inhibited leukemia cells growth, respectively. While hypoxia is a known factor mediating chemoresistance in solid tumors, our data presented here for the first time indicate the relevance of these processes in leukemia pathophysiology.

Various approaches have been explored to target the hypoxic microenvironment and thus render tumor cells susceptible to chemotherapy. Some approaches seek to directly inhibit HIF-1α activity, while others take advantage of the hypoxic microenvironment via hypoxia-activated prodrugs. In this study we used PR-104, a nitrogen mustard that under very low oxygen concentrations is reduced to its amine and hydroxyl amine metabolites which function as alkylating agents, leading to cell death. In vitro, the alcohol form of PR-104 had good cytotoxic activity against B-lineage ALL cell lines REH and Nalm6 and primary ALL cells. As expected, the cell killing effect was more pronounced in hypoxic compared to normoxic conditions. In cord blood cells used here as normal controls, the drug caused little cell death. The oxygen concentration required for 50% inhibition of PR-104A cytotoxicity in SiHa cells is only 0.1 µM, corresponding to 0.01% oxygen in the gas phase [52]. The true oxygen concentration in cells with 1% oxygen in the gas phase is unknown, but is probably not low enough to fully activate PR-104A. However, given that PIM and PR-104A are both nitro compounds, and probably have broadly similar oxygen dependencies, we tested PIM activation in Nalm6 and REH under decreasing oxygen concentrations and observed hypoxia dependent binding at 1%O_2_ and lower (Figure S1). These findings suggest that PR-104 could similarly, although not fully, be activated at 1% oxygen and that therefore the *in vitro* activity probably underestimates activity in severely hypoxic tissues.

Our results *in vitro* prompted us to test PR-104 in several in *vivo* leukemia models, where it showed remarkable antitumor activity as a single agent. Murine xenograft models harboring Nalm6-Luciferase ALL or primary ALL treated with PR-104 showed responses with significant decreases in the percentage of circulating CD45^+^ cells and prolongation of survival. Furthermore, the agent is likely effective against a broad range of acute leukemias as PR-104 was effective in reducing tumor burden when primary AML cells were used in the murine xenograft model. There were however differences in the overall responses to the treatment since mice injected with the aggressive MLL-ALL primary cells exhibited a gradual increase in circulating leukemia cells once the treatment was stopped, an event that was not observed in mice injected with primary AML.

Dose response curves were also evaluated in two xenograft models of primary ALL. PR-104 significantly delayed the progression of ALL-8 in a dose-dependent manner at all doses tested. Treatment at the three highest doses of this drug resulted in an objective response measure (ORM) of maintained complete response (MCR). Two of these three doses correspond to the plasma levels of the drug that can be achieved in humans; recent comparison of plasma pharmacokinetics has shown that the mouse dose equivalent to the MTD in humans with solid tumors (1100 mg/m^2^) is ∼125 mg/kg [31]. PR-104 also significantly delayed the progression of ALL-19 in a dose-dependent manner at the three highest doses tested. However, only the highest dose, which leads to plasma drug concentrations well above those achievable in the clinic, resulted in an ORM of MCR suggesting that combination therapies are needed. Since both ALL-8 and ALL-19 are derived from patients who experienced early relapse (within 2 years of diagnosis), possible differences in leukemia subtype specificity of PR-104 between T-lineage ALL (ALL-8) and B-cell-precursor ALL (ALL-19) should be explored in future studies. Interestingly, given the remarkable antitumor activity of PR-104, even at concentrations 50% or lower than the MTD, there seems to be higher activity of the agent in the leukemia models than that observed in the pediatric [35], [53] or adult [28] solid tumours. The reasons for the observed differences are not clear at this point and would not appear to simply relate to AKR1C3 expression levels, since the levels in the 6 solid tumors varied from as high as the T-ALL xenograft which showed the highest sensitivity to PR-104, to very low levels of expression. Therefore, one could speculate that it might relate to: (1) the known sensitivity of ALL to alkylating agents e.g. cyclophosphamide; (2) the homologous recombination repair status of the cells; (3) the difference in models tested (systemic model of leukemia versus subcutaneous solid tumors) and differences in PR-104 biodistribution that might impinge on this; (4) other unknown factors intrinsic to the different tumor types. Notably, another hypoxia-activated alkylating agent TH-302 exhibited remarkable anti-tumor effects on multiple disease parameters in a multiple myeloma model [40].

In all three models tested here, restoration of normal hematopoiesis resulted in drastic decline in the areas of PIM positivity possibly indicating normalization of BM vasculature and restoration of higher physiologic oxygen tension. It is conceivable to hypothesize that application of hypoxia-activated prodrugs would selectively eliminate leukemic cells in most hypoxic areas. We also propose that leukemic stem cells may have a propensity similar to HSC to reside in these areas resulting in the emergence of resistant clones. While these hypotheses require further experimental proof, they may develop into rational combinations of PR-104 with chemotherapeutic or other targeted agents. Another attractive approach would be the utilization of this class of drugs in the setting of minimal residual disease. However, given the recent finding that normal long-term repopulating cells may likewise reside in these areas, their elimination may result in prolonged myelosuppression. Indeed, myelosupression has been reported for PR-104 in a Phase I clinical trial in solid tumors [31]. It is arguable however whether this side effect is attributable to the ability of PR104 to target human progenitor cells residing in hypoxic areas, or to the fact that CD34 positive progenitors express AKR1C3, the enzyme responsible for aerobic activation of PR-104 [35]. In this respect, PR104-A indeed caused significant inhibition of the clonogenic survival of human progenitor cells in vitro, which was in turn ameliorated by AKR1C3 inhibitor naproxen (personal communications, J. Down and K. Parmar). Further studies with more selective hypoxia-activated prodrugs or HIF inhibitors will likely yield the answer to this clinically relevant question.

In summary, we have shown for the first time that the leukemic BM microenvironment is hypoxic and provided a rationale for targeting it. Our results with the bioreductive drug PR-104 suggest that targeting hypoxia is feasible and could have significant impact in the treatment of leukemias. Several *in vivo* murine leukemia models validated hypoxia as a potential target; as a consequence, PR-104 is undergoing a Phase I clinical trial in patients with relapsed and refractory AML and ALL. In the future, the ultimate goal would be to use bioreductive drugs in combination with conventional chemotherapies or novel targeted agents to eradicate resistant leukemic blasts that persist in the hypoxic BM microenvironment.

## Materials and Methods

### Chemicals, antibodies, and reagents

PR-104 and PR-104A were provided by Proacta. Anti-human AKR1C3 and anti-β-actin mAb were purchased from Sigma-Aldrich. Secondary antibodies IRDye 800CW Donkey anti-mouse and IRDye 680CW Donkey anti-mouse were obtained from LI-COR Biosciences. The following antibodies were used for IHC analysis: anti-GFP (Santa Cruz Biotechnology), anti-HIF-1α (BD Biosciences), FITC-conjugated mouse monoclonal anti-PIM (HPI, Inc), anti-CD45 (Cell Signaling Technology), anti-CD22 (Novocastra), anti-CXCR4 (Abcam), anti-SDF-1α, anti-CD31 (Santa Cruz Biotechnology) and anti-CAIX (Novus Biologicals). HRP conjugated secondary antibodies were from Dako (LSAB+system+HRP).

### Cell culture

REH and Nalm6 cells were purchased from American Type Tissue Collection. Nalm6-GFP-luciferase labeled cells were made at St Jude Children's Research Hospital by transducing Nalm6 cells with a murine stem cell virus-internal ribosome entry site-GFP retroviral vector containing the firefly luciferase gene. BM or peripheral blood samples were obtained for *in vitro* studies from patients with AML or ALL. Samples were collected during routine diagnostic procedures after written informed consent was obtained; protocols for studies in humans were approved by the Human Subjects Committee of The University of Texas MD Anderson Cancer Center. Mononuclear cells were separated by Ficoll-Hypaque (Sigma-Aldrich) density gradient centrifugation. Cell lines and primary samples were maintained in RPMI 1640 containing 10% FCS (Gemini Bio-Products) and 1% penicillin-streptomycin (Life Technologies Laboratories). Summarized patient characteristics are shown in Table S2.

Standard laboratory (normoxic) conditions comprised 21% O_2_ and 5% CO_2_ at 37°C. For experiments in a reduced oxygen environment, the hypoxic Workstation INVIVO2 400 from Ruskinn Technology was used. Cells were incubated in 1% O_2_ and 5% CO_2_ at 37°C.

### Immunohistochemistry

Human BM or mouse organs were harvested and fixed by immersion in 4% PFA. The use of human samples was approved by IRB and mouse samples by MDACC IACUC (ACUF Protocol # 01-06-01032). Sections (5 µm thick) were stained with H&E (X5000; Sigma-Aldrich) and analyzed by light microscopy. For the different staining of mouse tissue, the sections were incubated for 1 hour in blocking solution (PBS, 0.5% Tween-20, 0.1% BSA) and 10% FBS followed by incubation overnight with primary antibody. After washing, sections were incubated for 1 hour with secondary antibody, washed in PBS three times, and coverslips were mounted with Fluoromount-G (Electron Microscope Science). For human BM staining, formalin-fixed, paraffin-embedded tissue sections of BM biopsy specimens were deparaffinized in xylene (3 times for 5 minutes) and then rehydrated through graded concentrations of alcohol. For antigen retrieval, tissue sections were heated in EDTA (Biocare Medical, Concord, Calif) for 30 minutes in a Decloaking Chamber (Biocare Medical, Concord, Calif). Tissue sections were incubated with Biocare Medical peroxidase blocking reagent for 5 minutes. After washing with buffer, tissue sections were incubated with the primary antibodies for 60 minutes at room temperature (20°C). Sections were again washed, and incubated with secondary antibodies using Mach 4 AP Universal Polymer kit (Biocare Medical) according to manufacturer's instructions. For visualization, Vulcan Fast Red chromogen (Biocare Medical) was used for 10 minutes. All sections were counterstained with hematoxylin (Dako, Carpintera, Calif) for 3 minutes. Slides were then air dried and cover-slipped. Slides were analyzed under a 60x/1.40 PlanApo objective lens on an Olympus FV500 confocal microscope with Fluoview version 4.3 software (Olympus). For quantification of HIF-1α expression or PIM, at least 10 images/sample were aquired using the Nuance FX multispectral imaging system (CRi) which utilizes an optimized high-throughput tunable filter which has been matched to the bandwidths of common chromogens and fluorophores. Images were next quantified and scored by inForm software (CRi). The Nuance FX multispectral imaging system (CRi) was also used to generate pseudofluorescent images of DAB based IHC (PIM and HIF-1α). For assessment of bone marrow vasculature, slides were stained with DAPI and lectin was detected by TRITC fluorescence using the CRi multispectral imaging system. To quantify microvessel density (MVD), 10 random fields at 200× final magnification were examined for each condition (three mice each, NBM and bcCML) and the number of lectin positive microvessels per field was counted.

### PR-104A in vitro studies

Cells were seeded at 1×10^6^ cells/mL, treated with PR-104A at a final concentration of 5, 10, 15, or 25 µM, and incubated in normoxic (21%O_2_) or hypoxic conditions (1%O_2_) for 6 hours. The medium was then replaced with fresh medium, and cells were incubated in normoxic conditions until collection for FACS analysis.

### Cell death assessment

After appropriate treatment, cells were washed twice in PBS and resuspended in 100 µL Annexin binding buffer containing a 1∶100 dilution of Annexin V–FLUOS (Roche Applied Science). Cell numbers were quantitated after the addition of 10,000 CountBright counting beads (Invitrogen) per sample. Cells were then analyzed by a FACS Calibur flow cytometer (BD Biosciences) using 488-nm argon ion and 633-nm HeNe excitation lasers. For primary ALL samples, results were expressed as percentage of specific apoptosis calculated as: (% AnnV positive cells sample-% AnnV positive cells control)/(100-%AnnV positive cells control)*100.

### Western blot analysis

Cells were subjected to lysis in buffer supplemented with a protease inhibitor cocktail (Roche Diagnostics). Lysates were then separated on a 10% to 12% polyacrylamide gel, transferred to PVDF membranes (Life Science Research; Bio-Rad), probed with appropriate antibodies, visualized by using an enhanced chemiluminescence plus kit (GE Healthcare), and analyzed on the Odyssey imaging system from LI-COR Biosciences.

### Hypoxia measurement in BM flushes

BM flushes from PIM-administered mice were fixed in 4% PFA for 45 minutes at 4°C. After three washes with PBS, specimens were blocked overnight at 4°C in 0.3% Tween20, 1.5% BSA, and 5% mouse serum in PBS. Samples were stained overnight at 4°C with FITC-conjugated mouse mAb anti-pimonidazole (HPI, Inc) at 75 µg/mL. After two washes with 0.3% Tween-20 in PBS and one with PBS, specimens were resuspended in 1% PFA and analyzed by FACS. Results are expressed in terms of MFI.

### Animal studies

All animal work was done in accordance with a protocol approved by the institutional animal care and use committee of MD Anderson Cancer Center or University of New South Wales. Three-month-old NSG mice were irradiated with 300 cGy and injected with leukemic cells from an infant with MLL-rearranged B-lineage ALL (5×10^4^ viable cells per mouse). PR-104 treatment started 10 days after injection of cells under the following regimen: 200 mg/kg i.p., 3 days per week for 2 weeks. After 3 weeks without treatment, treatment was resumed at the same dose, twice a week for 2 weeks.

For the blast crisis CML model, C57Bl6/J mice were injected with 4×10^6^ murine HSC expressing the oncogenes *BCR/ABL* and *Nup98* and labeled with GFP/YFP [30]. Mice were humanly sacrificed 20 hours, 3 days, or 6 days after cell injection.

For the Nalm6 B-cell ALL model, NSG mice were injected i.v. with 1×10^6^ Nalm6-Luciferase cells/mouse. Starting on day 3, PR-104 at 250 mg/kg or PBS was administered i.p. 3 times a week for 2 weeks. Leukemia progression was evaluated weekly by luciferase imaging weekly as described elsewhere [54].

For the dose-response experiments, NOD/SCID mice were inoculated one of two ALL xenografts (ALL-8 and ALL-19) [34]. PR-104 was administered i.p. at doses of 550 mg/kg, 200 mg/kg, 100 mg/kg, or 50 mg/kg once a week for 6 weeks (seven mice per treatment group). Treatment commenced when the percentage of human CD45^+^ cells in peripheral blood exceeded the median value of 1% for the complete cohort. The experimental endpoint for each mouse was reached when the percentage of human CD45^+^ cells in peripheral blood reached 25%. The study was continued until all mice from the cohort reached this leukemia-related event or were sacrificed. Mice were sacrificed if they became morbid or had a weight loss of 20% or greater. Both event free survival (EFS) and leukemia growth delay (LGD) estimations were carried out according to established methodology. Individual mice were assigned a clinical “score” depending on the leukemic growth characteristics observed in the 42 days following treatment initiation according to the established criteria used for evaluating single agents [34], [35].

For the primary refractory FLT3-mutated AML model, NSG mice were injected i.v. with 20×10^6^ cells/mouse after irradiation (2.5 Gy). Starting on day 72 after cell injection, five mice were treated with PR-104 250 mg/kg or with PBS i.p. 3 times a week for 2 weeks. Cell engraftment and leukemia progression were measured by determining the percentage of CD45^+^ cells in peripheral blood by FACS.

### Detection of hypoxia in BM

Bones were collected from mice 3 hours after they received PIM 100 mg/kg i.p. and were processed for IHC.

### Detection of microvessel density in BM

TRITC conjugated Lectin from Ulex europaeus (SIGMA-ALDRICH) was injected into mice at 0.5 mg//mouse/IV 1 hour prior to sacrifice. Bones were collected and processed for IHC.

### Statistics

Unless otherwise indicated, results are expressed as mean ± SD of three independent experiments. *P*-values were determined by one-way ANOVA followed by F statistics or paired t test (when comparing two groups). A *P*-value less than 0.05 is considered significant. EFS between control and treated mice were compared by logrank test.

## Supporting Information

Figure S1 **Pimonidazole binding to Nalm6 and REH cell lines in a hypoxia dependent manner.** Cells were incubated with 100 µM PIM for 3 hours at different oxygen concentrations and then washed, fixed and processed for FACS. Control, cells stained with antibody only without PIM pre-incubation. MFI ratios compared to control were as follows: Nalm6 21% O_2_ 1.9; Nalm6 1%O_2_ 6.9; Nalm6 0%O_2_ 9.5; REH 21% O_2_ 1.6; REH 1%O_2_ 10.3; REH 0%O_2_ 22.2_._
(TIF)

Figure S2 **BM vasculature is altered in a syngenic model of blastic phase CML.** 1×10^7^ GFP/YFP labeled cells expressing the oncogenes *BCR/ABL* and *Nup98* were FACS-sorted and transplanted into irradiated (4.5GY) C57B16/J mice. Six days after cell transplantation, mice were injected with TRITC conjugated lectin 1 hr prior to sacrifice. Micro vessels were detected by fluorescence in BM from control leukemia-free mice (NBM) or bcCML bearing mice (bcCML). The lower panel shows quantification of lectin-positive vessels by CRi spectral imaging and Inform software analysis (3 mice/each, at least 10 slides per mouse were analyzed). Original magnification is shown on the figures. ***P<0.0001.
(TIF)

Figure S3 **A. HIF-1α is expressed at low levels in normal BM samples.** Representative images from 3 normal donors are shown. Original magnification, ×500. (HIF-1α: green; nuclei: red). B. HIF-1α is expressed in stromal cells as well as in leukemic blasts in BM biopsy from ALL patients. A representative image from one ALL BM sample obtained at diagnosis (4 samples were analyzed with similar findings). HIF-1α was detected by IHC and the different cells were identified based on morphology. Original magnification ×1000.
(TIF)

Figure S4 **AKR1C3 expression in cell lines (A), primary ALL samples (B), CD34^+^ cells from normal donors and cord blood (C) was detected by Western Blot.** Extract from SKOV3 cells was used as positive control.
(TIF)

Figure S5 **PR-104 decreased leukemia burden of NSG mice transplanted with primary refractory FLT3-mutated AML and restored tissue architecture.** Treatment started on day 72 after cell injection: 250 mg/kg, i.p. 3 times a week for 2 weeks (A) PBS (upper panel) or PR-104 (lower panel) treated mice were bled periodically and leukemia progression was assessed by determining the percentage of circulating human CD45 positive cells by FACS. X axis represents time with respect to starting of the treatment. (B) PR-104 inhibited AML leukemia growth in NSG mice in the lung, liver, bone, and spleen and resulted in dramatic reduction of hypoxic expansion observed in the control mice. CD45 and H&E IHC in lung, liver, BM, and spleen from PBS-treated control and PR-104-treated mice. (C) Pimonidazole was administered 3 hours prior to sacrifice and its adducts were detected in BM sections. Original magnification, ×400.
(TIF)

Table S1 **LGD summary and objective response measures (ORM) for PR-104-treated ALL-8 or ALL-19 engrafted mice.**
(DOC)

Table S2 **Primary patient data summary.** N/A: not available.
(DOC)
