# Supplementary figures and images for: Pronounced Hypoxia in Models of Murine and Human Leukemia: High Efficacy of Hypoxia-Activated Prodrug PR-104
Source: PLoS One. 2011 Aug 11;6(8):e23108. doi: 10.1371/journal.pone.0023108 (PMC3154919; doi:10.1371/journal.pone.0023108)

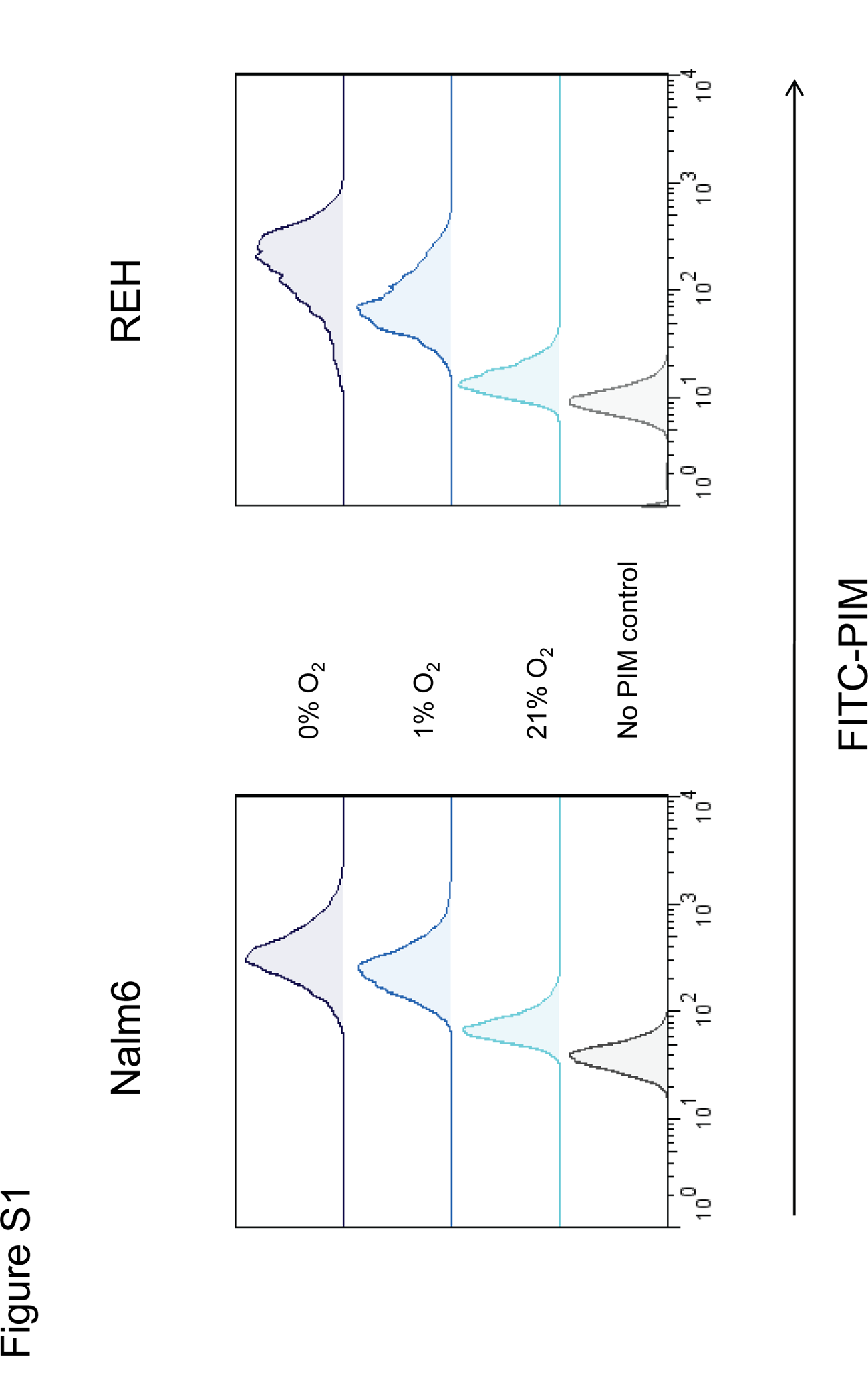

Supplement: Figure S1 — Pimonidazole binding to Nalm6 and REH cell lines in a hypoxia dependent manner. Cells were incubated with 100 µM PIM for 3 hours at different oxygen concentrations and then washed, fixed and processed for FACS. Control, cells stained with antibody only without PIM pre-incubation. MFI ratios compared to control were as follows: Nalm6 21% O2 1.9; Nalm6 1%O2 6.9; Nalm6 0%O2 9.5; REH 21% O2 1.6; REH 1%O2 10.3; REH 0%O2 22.2. (TIF) [file pone.0023108.s001.tif]

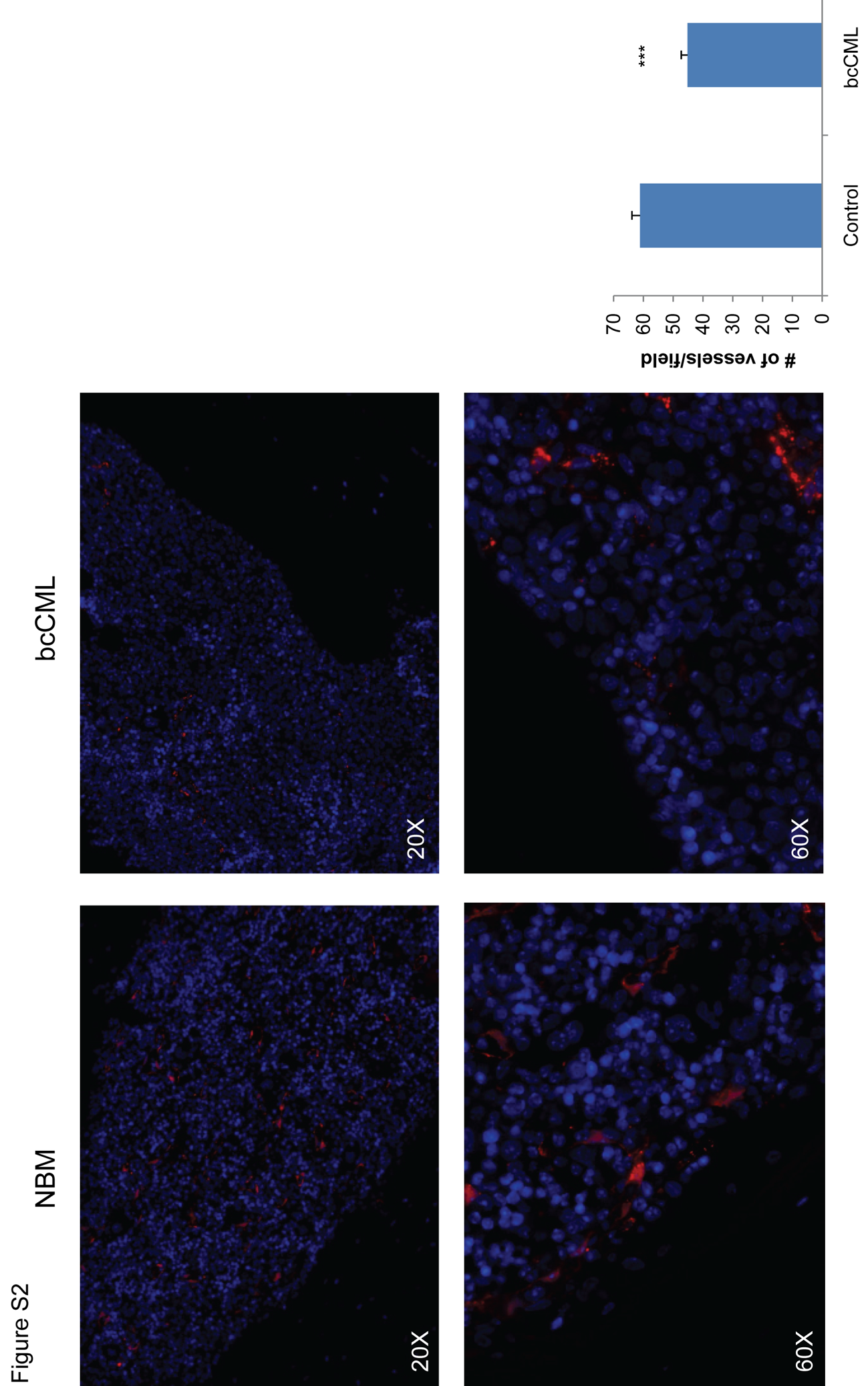

Supplement: Figure S2 — BM vasculature is altered in a syngenic model of blastic phase CML. 1×10^7^ GFP/YFP labeled cells expressing the oncogenes BCR/ABL and Nup98 were FACS-sorted and transplanted into irradiated (4.5GY) C57B16/J mice. Six days after cell transplantation, mice were injected with TRITC conjugated lectin 1 hr prior to sacrifice. Micro vessels were detected by fluorescence in BM from control leukemia-free mice (NBM) or bcCML bearing mice (bcCML). The lower panel shows quantification of lectin-positive vessels by CRi spectral imaging and Inform software analysis (3 mice/each, at least 10 slides per mouse were analyzed). Original magnification is shown on the figures. ***P<0.0001. (TIF) [file pone.0023108.s002.tif]

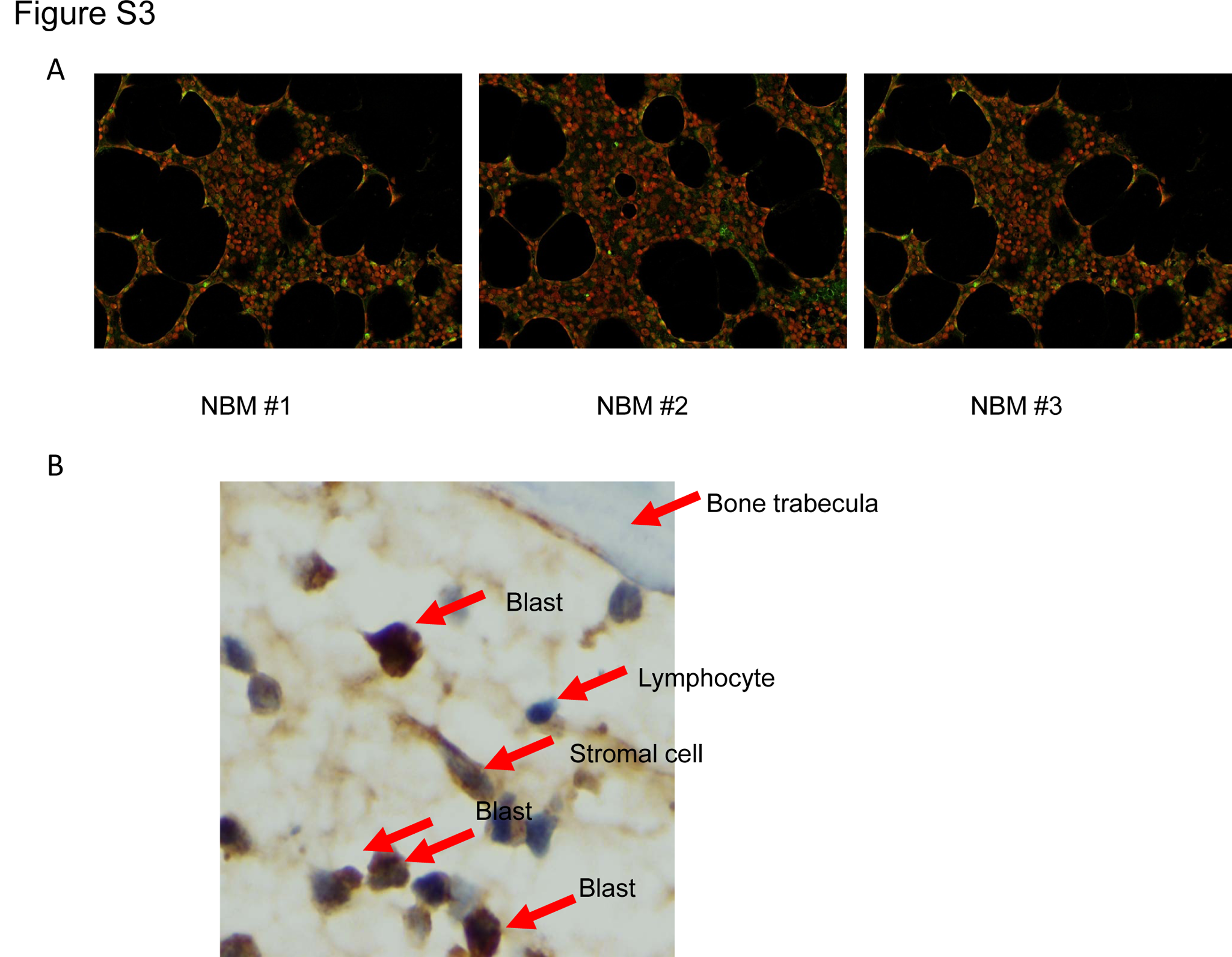

Supplement: Figure S3 — A. HIF-1α is expressed at low levels in normal BM samples. Representative images from 3 normal donors are shown. Original magnification, ×500. (HIF-1α: green; nuclei: red). B. HIF-1α is expressed in stromal cells as well as in leukemic blasts in BM biopsy from ALL patients. A representative image from one ALL BM sample obtained at diagnosis (4 samples were analyzed with similar findings). HIF-1α was detected by IHC and the different cells were identified based on morphology. Original magnification ×1000. (TIF) [file pone.0023108.s003.tif]

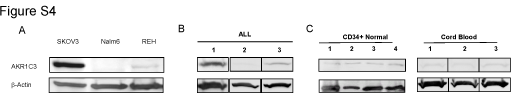

Supplement: Figure S4 — AKR1C3 expression in cell lines (A), primary ALL samples (B), CD34+ cells from normal donors and cord blood (C) was detected by Western Blot. Extract from SKOV3 cells was used as positive control. (TIF) [file pone.0023108.s004.tif]

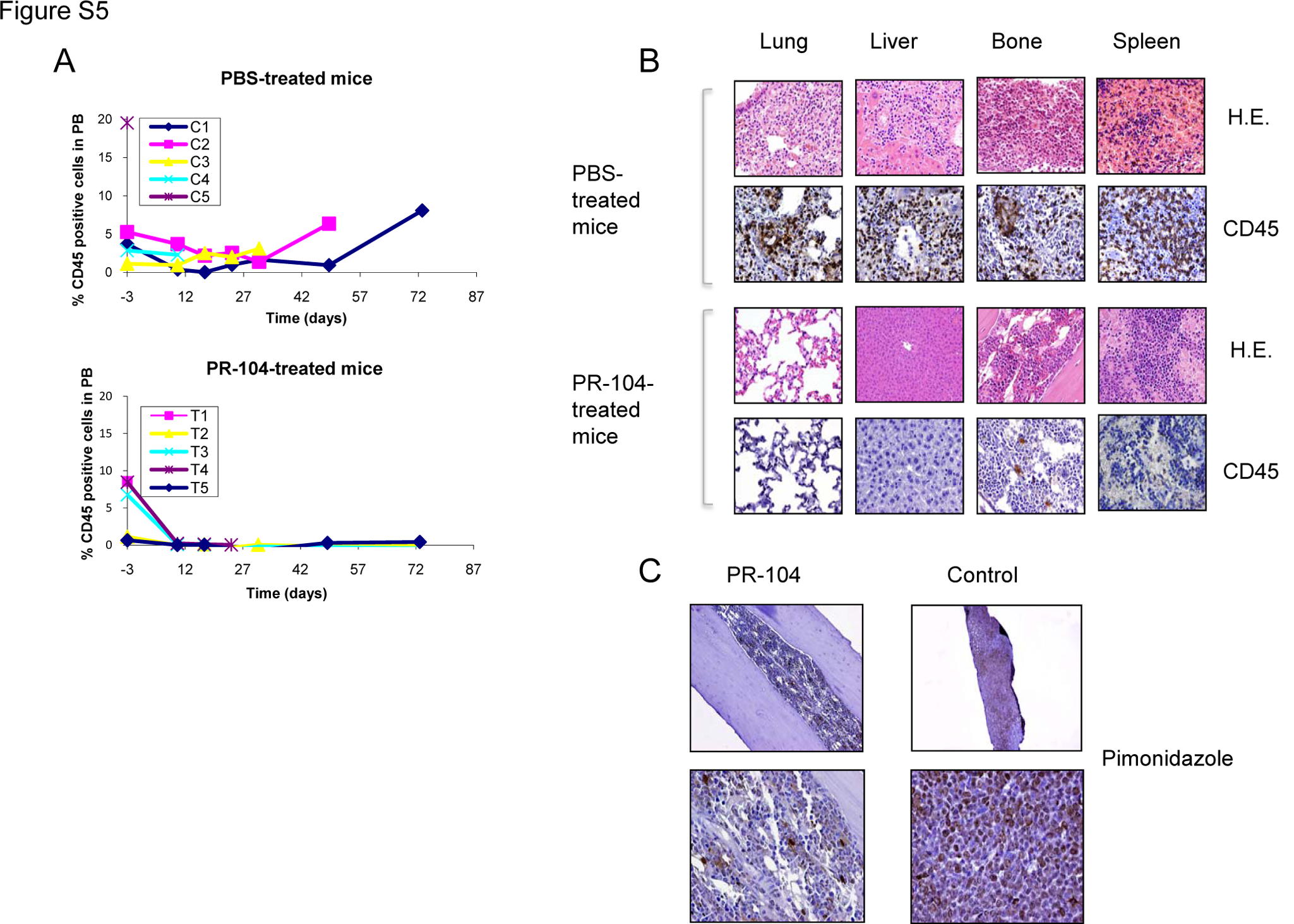

Supplement: Figure S5 — PR-104 decreased leukemia burden of NSG mice transplanted with primary refractory FLT3-mutated AML and restored tissue architecture. Treatment started on day 72 after cell injection: 250 mg/kg, i.p. 3 times a week for 2 weeks (A) PBS (upper panel) or PR-104 (lower panel) treated mice were bled periodically and leukemia progression was assessed by determining the percentage of circulating human CD45 positive cells by FACS. X axis represents time with respect to starting of the treatment. (B) PR-104 inhibited AML leukemia growth in NSG mice in the lung, liver, bone, and spleen and resulted in dramatic reduction of hypoxic expansion observed in the control mice. CD45 and H&E IHC in lung, liver, BM, and spleen from PBS-treated control and PR-104-treated mice. (C) Pimonidazole was administered 3 hours prior to sacrifice and its adducts were detected in BM sections. Original magnification, ×400. (TIF) [file pone.0023108.s005.tif]
