# Supplementary material for: Pronounced Hypoxia in Models of Murine and Human Leukemia: High Efficacy of Hypoxia-Activated Prodrug PR-104
Source: PLoS One. 2011 Aug 11;6(8):e23108. doi: 10.1371/journal.pone.0023108 (PMC3154919; doi:10.1371/journal.pone.0023108)
Supplement: Table S1 — LGD summary and objective response measures (ORM) for PR-104-treated ALL-8 or ALL-19 engrafted mice. (DOC) [file pone.0023108.s006.doc]

**Table S1. LGD summary and objective response measures (ORM) for PR-104-treated ALL-8 or ALL-19 engrafted mice.**

| **ALL-8** | **Median EFS** | **LGD** | **Significance** | **Median** |
| --- | --- | --- | --- | --- |
|  | **[days]** | **[days]** | **[p value]** | **ORM** |
| **Control** | **6.3** | **-** | **-** | **-** |
| **PR104 550 mg/kg** | **107.2** | **100.9** | **0.0001** | **MCR** |
| **PR104 200 mg/kg** | **88.8** | **82.5** | **0.0011** | **MCR** |
| **PR104 100 mg/kg** | **74.6** | **68.3** | **0.0011** | **MCR** |
| **PR104 50 mg/kg** | **39.8** | **33.5** | **0.0001** | **SD** |

| **ALL-19** | **EFS** | **LGD** | **Significance** | **Median** |
| --- | --- | --- | --- | --- |
|  | **[days]** | **[days]** | **[p value]** | **ORM** |
| **Control** | **4.9** | **-** | **-** | **-** |
| **PR104 550 mg/kg** | **>59.5** | **>54.6** | **0.0007** | **MCR** |
| **PR104 200 mg/kg** | **43.4** | **38.5** | **0.0018** | **SD** |
| **PR104 100 mg/kg** | **22.5** | **17.6** | **0.0052** | **PD2** |
| **PR104 50 mg/kg** | **8.3** | **3.4** | **0.2322** | **PD1** |

LGD: leukemia growth delay; EFS, event free survival; MCR, maintained complete response; SD, stable disease; PD, progressive disease
