# Supplementary material for: Pronounced Hypoxia in Models of Murine and Human Leukemia: High Efficacy of Hypoxia-Activated Prodrug PR-104
Source: PLoS One. 2011 Aug 11;6(8):e23108. doi: 10.1371/journal.pone.0023108 (PMC3154919; doi:10.1371/journal.pone.0023108)
Supplement: Table S2 — Primary patient data summary. N/A: not available. (DOC) [file pone.0023108.s007.doc]

| **Patient #** | **Source** | **Assay** | **Age** | **Immunophenotype** | **% Blasts** | **CG ALL Group** | **FAB ALL** | **Sample status** |
| --- | --- | --- | --- | --- | --- | --- | --- | --- |
| 1 | BM | HIF-1α-IHC | 39 | Pre-B ALL | 60 | del(6q) | L1 | Newly diagnosed |
| 2 | BM | HIF-1α-IHC | 18 | Pre-B ALL | 84 | t(8;14), t(8;2) | L2 | Newly diagnosed |
| 3 | BM | HIF-1α-IHC | 61 | Pre-B ALL | 82 | Ph+ | L2 | Newly diagnosed |
| 4 | BM | HIF-1α-IHC | 58 | Pre-B ALL | 96 | t(4;11) | L2 | Newly diagnosed |
| 5 | BM | HIF-1α-IHC | 34 | Pre-B ALL | 92 | Insufficient Metaphases | L2 | Newly diagnosed |
| 6 | BM | HIF-1α-IHC | 21 | Pre-B ALL | 94 | +21 | L2 | Newly diagnosed |
| 7 | BM | HIF-1α-IHC | 56 | Pre-B ALL | 70 | +21 | L1 | Newly diagnosed |
| 8 | BM | HIF-1α-IHC | 33 | Pre-B ALL | 88 | t(4;11) | L2 | Newly diagnosed |
| 9 | BM | HIF-1α-IHC | 53 | Pre-B ALL | 90 | Insufficient Metaphases | N/A | Newly diagnosed |
| 10 | PB | PR-104 cytotoxicity | 29 | Pre-B ALL | 30 | Diploid | L2 | Newly diagnosed |
| 11 | PB | PR-104 cytotoxicity | 35 | T-ALL | 94 | del(6q), del(12p) | N/A | Newly diagnosed |
| 12 | PB | PR-104 cytotoxicity | 48 | Pre-B ALL | 67 | Diploid | L2 | Newly diagnosed |

Table S2. Primary patient data summary.

N/A: not available; IHC: immunohistochemistry.
